# Metagenomic profiling of pathogens and antibiotic resistome in influent of six municipal wastewater treatment plants: a descriptive analysis of plant-specific microbial hazards

**DOI:** 10.3389/fmicb.2026.1780611

**Published:** 2026-08-03

**Authors:** Peixi Qin, Waresi Tuersong, Zhuolin Tao, Bingyan Huang, Lili Tan, Hui Liu, Junlong Zhao, Min Hu

**Affiliations:** 1 National Key Laboratory of Agricultural Microbiology, College of Veterinary Medicine, Huazhong Agricultural University, Wuhan, China; 2 College of Veterinary Medicine, Xinjiang Agricultural University, Urumqi, China; 3 Guizhou Zhuxin Water Environment Co., Ltd, Guiyang, China

**Keywords:** antibiotic resistance genes (ARGs), pathogens, plant-specific hazards, urban microbiome, wastewater-based epidemiology

## Abstract

**Introduction:**

Wastewater treatment plants (WWTPs) serve as critical nodes for monitoring urban biological hazards, yet the raw influent—the primary entry point for pathogens and antibiotic resistance genes (ARGs)—remains less characterized compared to treated effluent, particularly at the level of individual facilities, as most prior studies have pooled samples or focused on post-treatment matrices.

**Methods:**

In this descriptive study, we performed metagenomic sequencing on influent samples collected from six municipal WWTPs, with each plant treated as an independent unit to profile its specific microbial community, pathogen composition, and antibiotic resistome.

**Results:**

Across all samples, a total of 853 bacterial and 232 eukaryotic pathogen species were identified. An exploratory risk index, calculated by integrating species abundance with established risk group classifications, assigned the highest heuristic score to Tangxun Lake (2150), reflecting its concurrent enrichment of both enteric and respiratory pathogens. The pathogen distribution exhibited plant-specific patterns: enteric pathogens including *Escherichia coli*, *Vibrio cholerae*, and *Campylobacter jejuni* were predominantly detected in Huangpu road and Nantaizi Lake, whereas respiratory pathogens such as *Mycobacterium tuberculosis* and *Legionella pneumophila* were more abundant in Xinzhuang, Jinyang, and Tangxun Lake. A core set of ARGs—comprising multidrug efflux pumps, *β*-lactamases, and tetracycline resistance genes—was consistently present across all six facilities, collectively accounting for approximately 60% of the total ARG abundance detected. In addition, exploratory correlations between mobile genetic elements (e.g., plasmids and transposases) and clinically relevant ARGs were observed across the dataset, warranting further investigation.

**Discussion:**

By generating plant-specific hazard inventories rather than pooled averages, this study provides a descriptive baseline that enables facility-specific surveillance prioritization.

## 1 Introduction

Rapid urbanization has established wastewater treatment plants (WWTPs) as essential bioreactors that consolidate multi-source waste streams, making them hotspots for pathogens and antibiotic resistance genes (ARGs) (Dubey et al., 2022; Zhu et al., 2025; Karaolia et al., 2021). The microbial community in the influent thus serves as a functional mirror of the urban microbiome, providing a valuable resource for assessing public health status (Cai and Zhang, 2013). While metagenomics has been extensively applied to study activated sludge and effluent microbiomes, the influent community—the primary source of pollutants and microorganisms entering the treatment plant—remains comparatively underexplored (Pandey et al., 2023; Zhou et al., 2019). The influent community structure directly influences the loading and potential risks imposed on downstream processes, a comprehensive characterization of this influent “input” is therefore fundamental for assessing and managing the risks associated with the final effluent “output” (Manaia et al., 2018).

In particular, a comprehensive assessment of health hazards posed by the co-occurrence of diverse pathogens and ARGs, considering their transmission routes (e.g., waterborne vs. aerosol), remains insufficient. Moreover, while numerous investigations have focused on microbial community structure and diversity, far less attention has been given to the systematic characterization of pathogen composition and the antibiotic resistome (Sun et al., 2023). This gap is concerning because influent wastewater contains a wide array of enteric, respiratory, and opportunistic pathogens that may pose risks to plant workers and downstream communities (Poopedi et al., 2025). Moreover, the presence of ARGs in influent, particularly those encoding resistance to clinically critical antibiotics, raises questions about their persistence and potential mobilization through the treatment process (Wirth et al., 2026). Understanding the baseline pathogen and ARG profiles at individual WWTPs is therefore a prerequisite for evaluating local exposure risks and designing targeted monitoring strategies.

A recent nationwide study on activated sludge from 28 A^2^O WWTPs in China, including Wuhan and Guiyang, revealed significant regional differentiation in ARG composition and highlighted the complex interplay of bacteria, viruses and environmental factors (Liu et al., 2026). However, that study focused on activated sludge—the post-treatment biological mixture—and on the multidimensional network of bacterial-viral-environmental interactions. In contrast, the present study focuses on influent (raw microbial input from urban catchments) and provides a purely descriptive, plant-by-plant characterization of pathogenic microorganisms and their associated ARGs, treating each WWTP as an independent unit. This study included six WWTPs located in two Chinese cities (Wuhan and Guiyang), generating plant-specific hazard inventories rather than pooled averages. To provide a descriptive baseline, we employed metagenomic sequencing to profile the influent microbiomes of these six WWTPs. Specifically, this study addresses three scientific questions: (1) What are the microbial community structures in each of the six WWTPs? (2) What are the pathogen compositions in each plant? (3) What are the ARG profiles in each of the six WWTPs?

## 2 Materials and methods

### 2.1 Collection of samples

**Figure 1:**
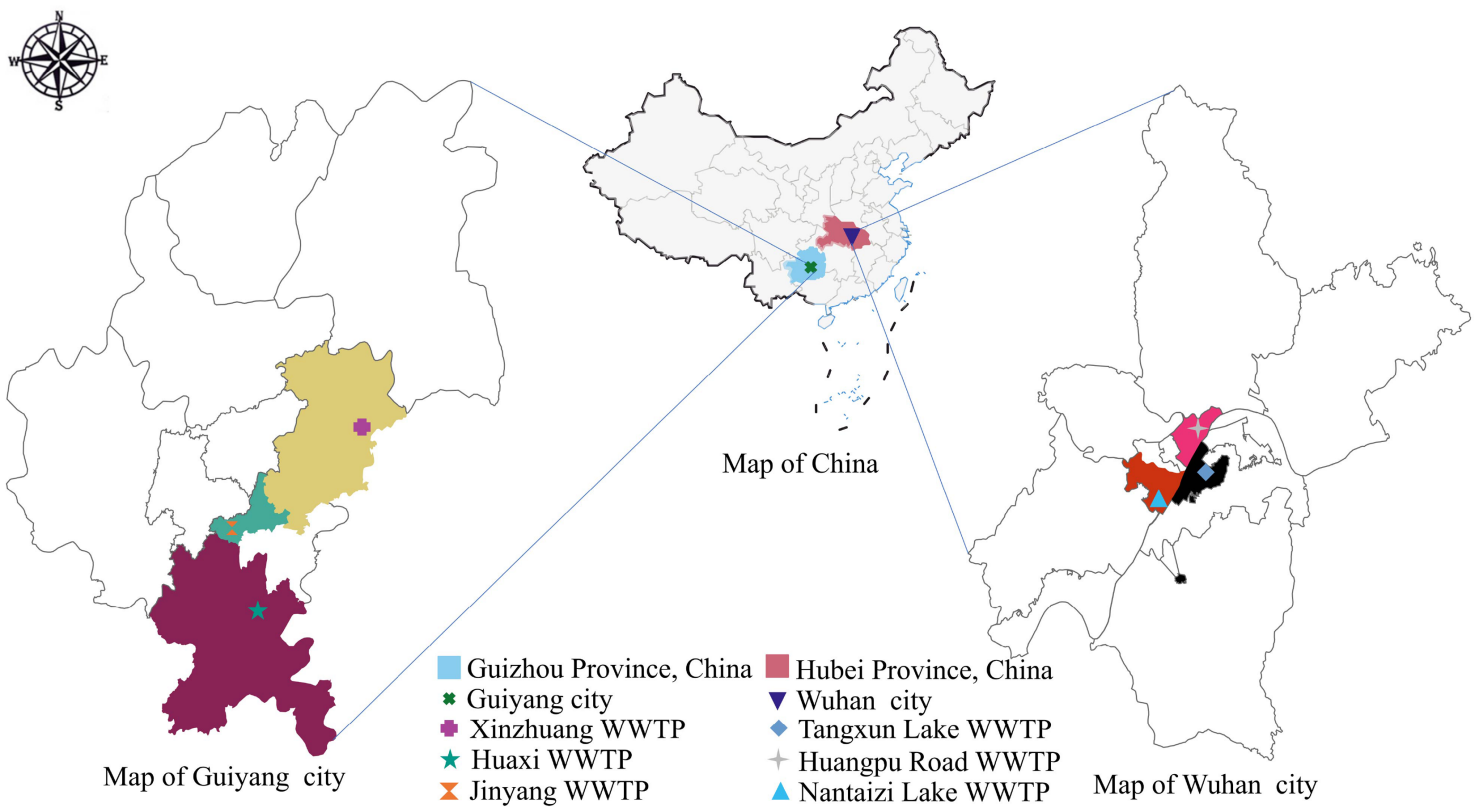
Sampling locations of the wastewater treatment plant in this study.

In October 2024, three 2 L influent samples were collected from each of the three WWTP in Wuhan, the capital of Hubei Province (29°58′–31°22′N, 113°41′–115°05′E): Tangxun Lake (TXH), Huangpu Road (HPL), and Nantaizi Lake (NTZH). In February 2025, three 2 L samples were collected from each of the three plants in Guiyang, the capital of Guizhou Province (26°11′–27°22′N, 106°07′–107°17′E): Xinzhuang (XZ), Huaxi (HX), and Jinyang (JY) (Figure 1). The two different sampling seasons (autumn 2024 in Wuhan and winter 2025 in Guiyang) were used to accommodate the project schedule and site access; each of the six WWTPs was treated as an independent sampling unit. All samples were kept at 4 °C from collection until processing, with transport and storage conducted under the same temperature condition, and processed within 24 h of collection.

### 2.2 Physicochemical analysis

Key physicochemical parameters of the influent wastewater, including chemical oxygen demand (COD), biochemical oxygen demand (BOD₅), ammonium nitrogen (NH_4_^+^-N), and total phosphorus (TP), were analyzed to characterize the influent wastewater quality. All parameters were measured in accordance with the Chinese standard methods (CJT51-2018). Specifically, COD was determined by the rapid sealed digestion method, BOD_5_ by the 5-day incubation method at 20 °C, NH_4_^+^-N by Nessler’s reagent spectrophotometry, and TP by ammonium molybdate spectrophotometry following persulfate digestion. The limit of detection (LOD), determined as three times the standard deviation of seven replicate blank measurements, was 4 mg/L for COD, 2 mg/L for BOD_5_, 0.02 mg/L for NH_4_^+^-N, and 0.03 mg/L for TP. All analyses were performed in triplicate, and the analytical precision, expressed as the relative standard deviation (RSD), was less than 2% for all measured parameters (Ministry of Housing and Urban-Rural Development of the People’s Republic of China, 2018).

### 2.3 Sample processing

Wastewater samples were centrifuged at 4,000 rpm for 30 min to collect the precipitate. The supernatant was then sequentially filtered through a series of membranes (pore sizes 2.7 μm to 0.22 μm) using a vacuum filtration system to enrich microbial cells, following standard methods (Ke et al., 2023). All collected materials, including precipitates and filter membranes, were transported to Personalbio (Shanghai, China) for metagenomic sequencing.

### 2.4 DNA extraction and quality control

Genomic DNA was extracted from the samples using the Mag-Bind® Soil DNA Kit (Product No. M5635-02; OMEGA Bio-Tek, USA) according to the manufacturer’s instructions. The concentration of the extracted DNA was quantified using a Qubit® 4 Fluorometer (Thermo Fisher Scientific, USA) with the dsDNA HS Assay Kit. DNA integrity was evaluated by 1% agarose gel electrophoresis, all DNA samples showed clear main bands without significant smearing, indicating high integrity. All DNA extracts were stored at −20 °C until further analysis.

### 2.5 Library preparation and sequencing

DNA libraries were prepared using the Illumina TruSeq® DNA Library Prep Kit (Illumina, USA) following the manufacturer’s protocol. Sequencing was performed on an Illumina NovaSeq 6000 platform (Illumina, USA), generating 150 bp paired-end reads.

### 2.6 Data preprocessing and taxonomic analysis

Raw sequencing reads in FASTQ format underwent quality control and preprocessing. Adapter sequences and low-quality bases were trimmed using Trimmomatic with the following parameters: SLIDINGWINDOW:4:20 and MINLEN:50. Sequence quality was assessed using FastQ, including the Q20 value (base call accuracy ≥99%), and reads with an average quality score below Q20 were discarded. Taxonomic classification was conducted with Kaiju (version 1.9.0) using the nr_euk database (downloaded on 02 November 2024 from)^1^, which contains non-redundant protein sequences from bacteria, archaea, fungi, and microbial eukaryotes. Kaiju was chosen because its protein-level alignment strategy provides high sensitivity for divergent taxa, and a recent wastewater-focused benchmark confirmed it as the accurate classifier at both genus and species levels (Di Gloria et al., 2025).

### 2.7 Identification of antibiotic resistance genes (ARGs)

Antibiotic resistance genes (ARGs) were identified and classified following the method described by Ali et al. (2021). Briefly, ARG-like sequences were extracted and classified using the ARGs-OAP v.2.0 software pipeline with the Structured Antibiotic Resistance Genes reference database version 2 (SARG v2.0). SARG v2.0 is a collection of 12,307 manually curated non-redundant ARG sequences derived from the ARDB, CARD, and NCBI-NR databases (NCBI-NR version dated 21 July 2016). The detection process began with a local pre-screening phase to extract potential ARG-like sequences using UBLAST searches against the SARG v2.0 database. The extracted ARG-like sequences were then annotated and classified using the online Galaxy instance of ARGs-OAP v2.0.^2^ ARG annotation was performed directly on quality-filtered raw reads using the ARGs-OAP pipeline, without prior assembly of reads into contigs. The annotation parameters were set as follows: E-value cutoff ≤1e^−5^, minimum sequence identity ≥90%, and minimum alignment length ≥25 amino acids. The abundance of each ARG was normalized to transcripts per million (TPM) based on the number of reads mapped to each ARG, to account for differences in sequencing depth across samples. The gene names reported in this study follow the hierarchical ‘type-subtype’ nomenclature of the SARG v2.0 database. For detailed functional descriptions, resistance mechanisms, and antibiotic class information of a specific gene, readers can consult the source databases: the Comprehensive Antibiotic Resistance Database (CARD)^3^ is recommended as the primary resource, as it provides the most extensive metadata, while the ARDB^4^ also offers relevant information.

### 2.8 Statistical analysis

**Figure 2:**
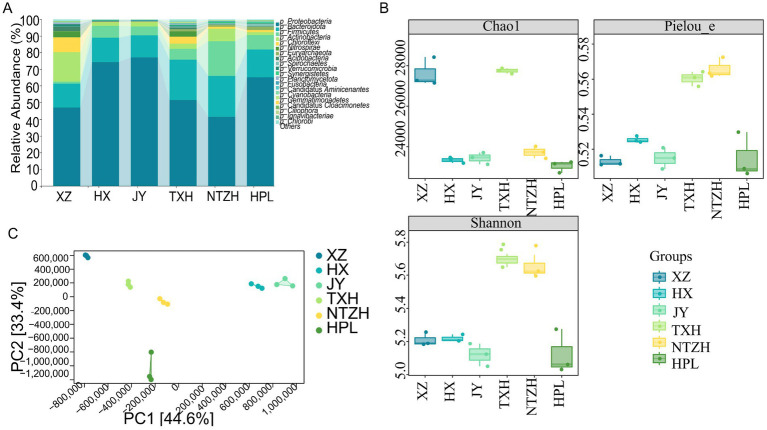
Microbial community structure and diversity in influent wastewater across six WWTPs. **(A)** Relative abundance of the dominant bacterial phyla (top 20). The bar plot shows the mean relative abundance (%) of major bacterial phyla across six plants. Each color represents a different phylum as indicated in the legend. Low-abundance phyla (<1% average relative abundance) were grouped as “Others.” **(B)** Alpha diversity indices (Chao1 richness, Pielou evenness, and Shannon diversity) for each WWTP. Each point represents a technical replicate (*n* = 3 per plant). **(C)** Principal component analysis (PCA) of microbial community composition based on Euclidean distance of relative abundance data. PC1 and PC2 explain 44.6 and 33.4% of the total variance, respectively. Each point represents an individual sample, color-coded by WWTP. Abbreviations: XZ, Xinzhuang; HX, Huaxi; JY, Jinyang; TXH, Tangxun Lake; NTZH, Nantaizi Lake; HPL, Huangpu Road. All analyses are descriptive; no statistical comparisons were performed among plants or regions.

**Figure 3:**
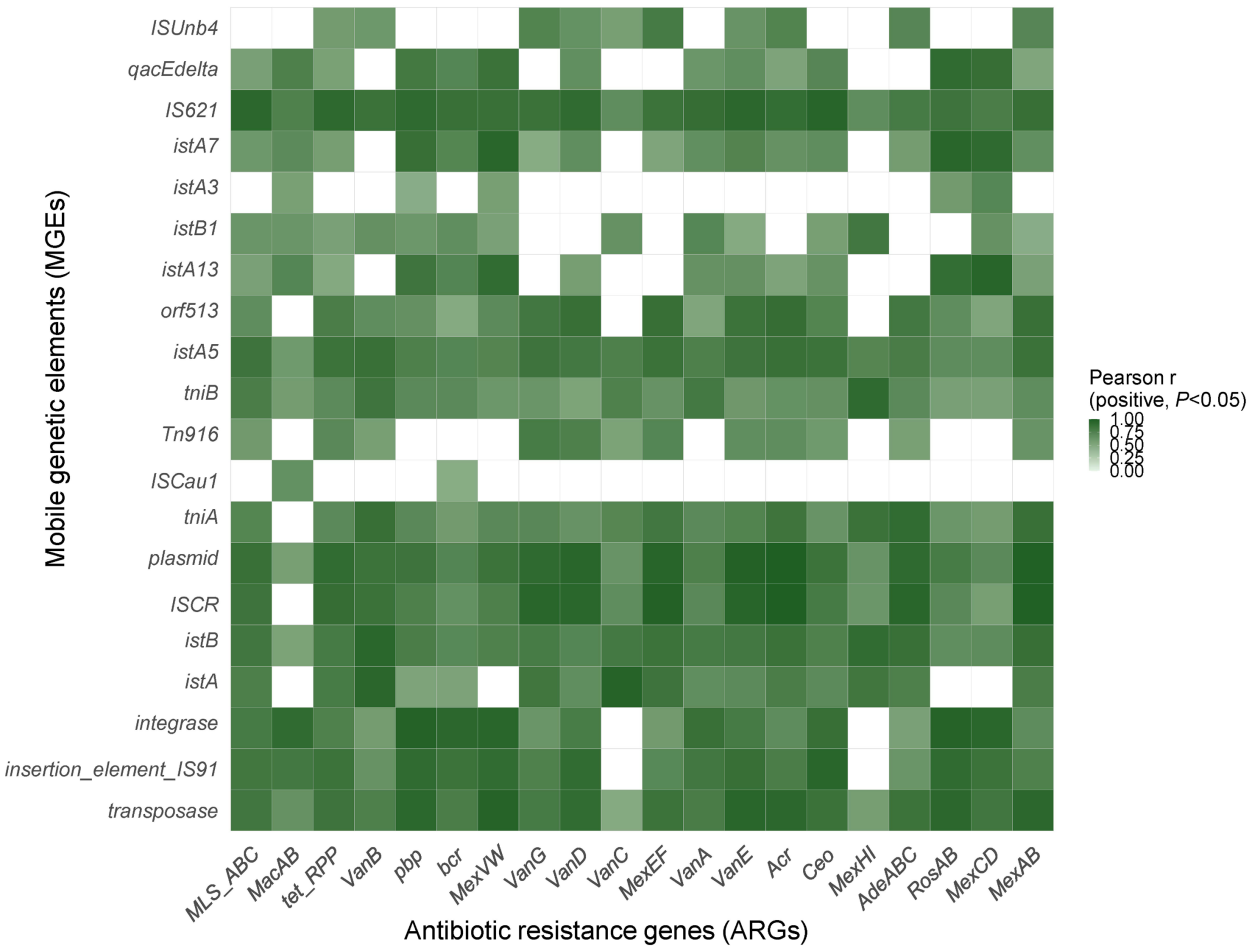
Correlation analysis between mobile genetic elements (MGEs) and antimicrobial resistance genes (ARGs). Pearson correlation coefficients based on log10-transformed raw read counts. To reduce skewness and satisfy the assumptions of Pearson correlation, raw read counts were log10-transformed before calculating correlation coefficients. Significant positive correlations (*p* < 0.05) are shown with a green gradient (darker = stronger). No significant negative correlations were found. White = not significant (*p* ≥ 0.05). Statistical caveats: no multiple-testing correction was applied; read-level co-abundance does not imply physical linkage.

Unit of replication. In this study, three grab samples were collected from the same location within each wastewater treatment plant (WWTP) on a single day. These three samples are technical replicates of the same plant condition, not independent biological or temporal replicates (Hurlbert, 1984). Therefore, the true independent replication unit is the WWTP itself (*n* = 6). Because the design does not provide independent replicates at the regional level, no cross-regional hypothesis testing was performed; all analyses are descriptive. Data normalization for microbial taxa. To account for differences in sequencing depth across samples, the raw Kaiju output (read counts per taxon) was converted to relative abundances by dividing each taxon’s read count by the total bacterial reads per sample. Alpha diversity. Microbial diversity within samples (alpha diversity) was assessed using the Chao1, observed features (i.e., the number of distinct species-level taxa detected per sample), and Shannon indices. To avoid pseudoreplication, we report the mean and range of these indices for each WWTP separately. No statistical comparisons were made among plants or regions. Beta diversity. Between-sample microbial community composition was visualized using principal component analysis (PCA) on the relative abundance data. PCA (based on Euclidean distance) was chosen for its simplicity and wide interpretability. We acknowledge the compositional nature of microbiome data; however, because no formal statistical test (e.g., PERMANOVA) was performed and the results are purely descriptive, the choice of distance metric does not affect any statistical inference. The PCA plot (Figure 2C) is presented for descriptive purposes only, and no formal test was applied. For the MGE-ARG correlation analysis (Figure 3), no multiple-testing correction was applied, and read-level co-abundance does not imply physical linkage or horizontal gene transfer.

### 2.9 Pathogen classification by affected system

Pathogens were categorized by their primary affected system (e.g., respiratory tract, gastrointestinal tract, multisystem) based on literature indexed in the NCBI PubMed/MEDLINE database and the MeSH (Medical Subject Headings) annotation.^5^ For each species, its known infection sites and associated disease manifestations were retrieved from the NCBI Taxonomy database^6^ and from relevant PubMed-indexed articles, including peer-reviewed reviews and case series. Species with documented ability to affect two or more organ systems were classified as “multisystem.”

### 2.10 Risk assessment

To obtain a unified numeric risk level (1, 2, or 3) for each detected bacterial species, we consulted three official classification sources in the following hierarchical order: (1) the Leibniz Institute DSMZ risk group database (based on EU Directive 2000/54/EC and German TRBA regulations), (2) the Chinese Catalogue of Pathogenic Microorganisms (2023 edition), and (3) the BMBL (6th edition) NIH risk group classification. When a species was listed in the DSMZ database, its DSMZ risk group (1, 2, or 3) was directly adopted. For species absent from DSMZ but present in the Chinese Catalogue, the Chinese hazard class was mapped to risk level 2 or 3 as follows: hazard class 2: risk level 2; hazard class 1: risk level 3 (as class 1 corresponds to RG4 in the EU system). For species absent from both DSMZ and the Chinese Catalogue but present in the BMBL risk group classification, the BMBL RG (2 or 3) was adopted. Species not listed in any of these three sources but with documented human infection cases in NCBI were assigned risk level 1. Species absent from all four sources (three directories plus NCBI case reports) were excluded from the risk assessment. This hierarchical rule ensures a deterministic, reproducible assignment of a unified 1–3 scale for downstream risk prioritization, acknowledging that the underlying definitions of these sources are not identical but are aligned in their core principle of ranking microorganisms by their potential hazard to human health. For each WWTP, the relative abundance of each species was converted into an abundance score (0: 0; 1: ≤0.1%; 2: 0.1–1%; 3: >1%). The top-30 species contributing most to the RI were identified per WWTP. The risk index (RI) for a plant was calculated as Σ (abundance score × RG). This risk index is an exploratory heuristic for internal comparison among the six WWTPs and has not been externally validated. It does not represent absolute risk. The top 30 species contributing most to the RI were identified per WWTP for descriptive ranking purposes.

The raw sequencing data have been deposited in the NCBI Sequence Read Archive (SRA) under the BioProject accession number PRJNA1330490.

Ethical statement: This study did not involve human participants or animal experiments.

## 3 Results

### 3.1 Physicochemical characteristics of WWTPs influents

**Figure 4:**
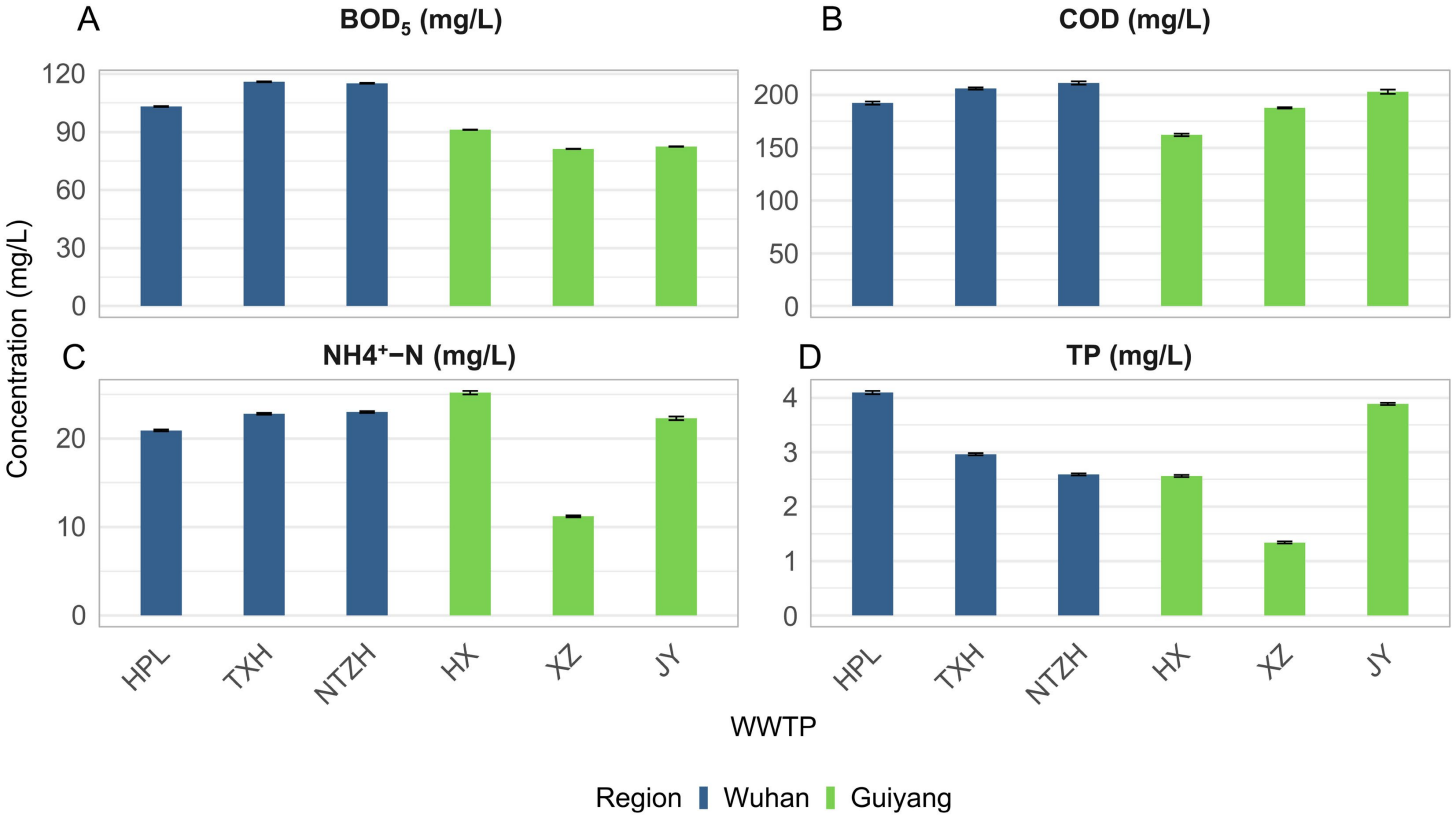
Physicochemical parameters of influent wastewater across six WWTPs. The multi-panel plot shows the mean concentrations (± standard deviation, error bars) of **(A)** five-day biochemical oxygen demand (BOD₅), **(B)** chemical oxygen demand (COD), **(C)** ammonium nitrogen (NH_4_^+^-N), and **(D)** total phosphorus (TP) for each of the six wastewater treatment plants (WWTPs). Sites are color-coded by region: Wuhan (HPL, TXH, NTZH) in blue-purple and Guiyang (HX, XZ, JY) in yellow-green. Abbreviations for sites: HPL, Huangpu Road WWTP; TXH, Tangxun Lake WWTP; NTZH, Nantaizi Lake WWTP; HX, Huaxi WWTP; XZ, Xinzhuang WWTP; JY, Jinyang WWTP.

Abbreviations used in this section: WWTPs: XZ, Xinzhuang; HX, Huaxi; JY, Jinyang; TXH, Tangxun Lake; NTZH, Nantaizi Lake; HPL, Huangpu road. Parameters: COD, chemical oxygen demand; BOD₅, five-day biochemical oxygen demand; NH_4_^+^-N, ammonium nitrogen; TP, total phosphorus. Figure 4 Shows the physicochemical characteristics of the influent wastewater. Notably, Wuhan WWTPs generally exhibited higher organic loading (COD, BOD_5_), while nutrient levels (NH_4_^+^-N, TP) varied markedly among plants, with the highest TP observed at HPL and the highest NH_4_^+^-N at HX. Detailed numerical data (means ± SD) are provided in Supplementary Table S1.

### 3.2 Overall microbial community structure and core taxa

A total of 18 influent wastewater samples were subjected to metagenomic sequencing, generating approximately 552 million raw paired-end reads. After quality filtering, 543.6 million PE high-quality reads (97.67–98.78% of raw data) were retained (Supplementary Table S2), demonstrating sufficient sequencing depth for comprehensive microbial community analysis.

Proteobacteria were the most abundant phylum in all six WWTPs (35.28–67.71%), followed by Bacteroidota (10.99–20.08%) and Firmicutes (0.98–17.89%). The complete relative abundance data for all phyla are provided in Supplementary Table S3 and Figure 2A. For visualization, only the top 20 most abundant phyla are shown in Figure 2A, with the remaining phyla grouped as ‘Others’; the relative abundances were normalized so that the displayed categories together sum to 100%. The complete abundance table at the species level, including all taxa and all individual technical replicates (18 samples), is provided in Supplementary Table S4 (TPM values). Alpha diversity indices (Shannon, Chao1, and Pielou’s evenness) were calculated for each technical replicate. For each WWTP, the three replicate values were averaged to obtain a single estimate per plant (*n* = 6). The Shannon index ranged from 5.12 ± 0.13 (HPL) to 5.69 ± 0.04 (TXH). Chao1 richness was highest in TXH (27,796 ± 87) and XZ (27,611 ± 622), and lowest in HPL (23,049 ± 226). Pielou’s evenness showed little variation among plants, with NTZH having the highest value (0.566 ± 0.006) (Figure 2B).

Beta diversity analysis showed that Principal Component 1 (PC1) and Principal Component 2 (PC2) explained 44.6 and 33.4% of the total variance, respectively. The Huangpu Road WWTP (HPL) was located at the extreme negative end of PC1, indicating a distinct microbial community structure compared to the other five WWTPs. This distinct clustering may be related to local operational factors, including the combined sewer system at this plant (Ali et al., 2021) (Figure 2C).

**Figure 5:**
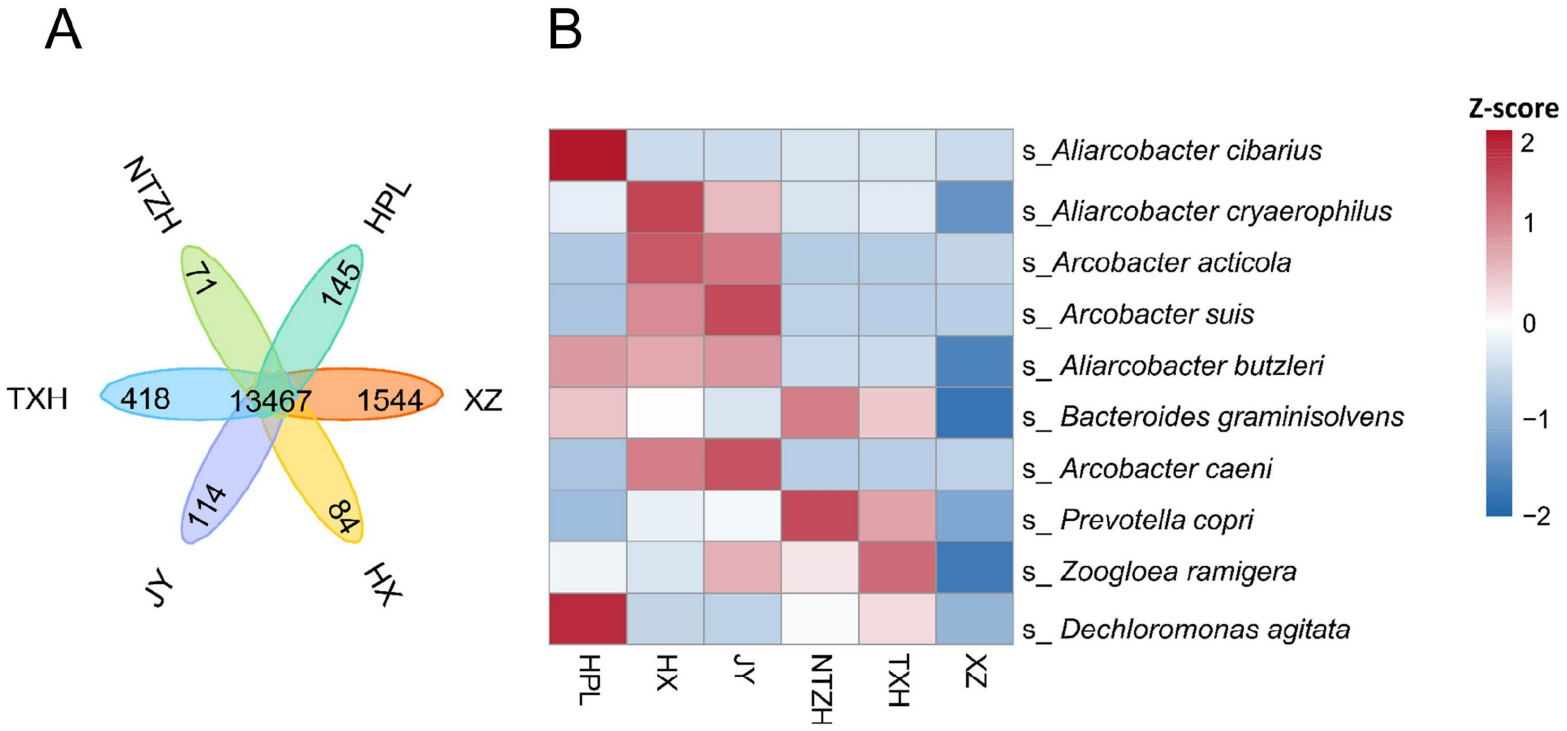
Group-wise comparison of microbial taxa across the six WWTPs. **(A)** Venn diagram showing the number of shared and unique microbial species among the six WWTPs. **(B)** Heatmap of the top 10 most abundant microbial species. For each WWTP, relative abundances from three technical replicates were averaged to obtain a plant-level value (*n* = 6). Species were ranked by their mean relative abundance across the six plants. Only species with complete binomial names (i.e., genus species without brackets, “Candidatus,” “Unclassified,” “bacterium,” or “sp.”) were considered. The abundance values (relative abundance, row Z-score normalized) are visualized using a blue-white-red color scale: red indicates that the abundance in that WWTP is higher than the species’ own six-plant mean, blue indicates lower than the mean, and white indicates close to the mean. WWTP abbreviations: XZ (Xinzhuang), HX (Huaxi), JY (Jinyang), TXH (Tangxun Lake), NTZH (Nantaizi Lake), HPL (Huangpu Road). No statistical tests were performed.

In the present study, a total of 15,843 microbial species were identified across the six WWTPs, with 13,467 core species shared among all facilities. The number of unique species in each WWTP was reported as follows: Xinzhuang WWTP (1,544), Huangpu Road WWTP (145), Nantaizi Lake WWTP (71), Tangxun Lake WWTP (418), and Jinyang WWTP (114) (Figure 5A). Additionally, the top 10 most abundant microbial species across the six WWTPs were identified by ranking the mean relative abundance of each species across the six plants (Figure 5B) (plant-level averages of three technical replicates, *n* = 6). Only species with complete binomial names (i.e., *s_Genus species* without brackets, “Candidatus,” “Unclassified,” “bacterium,” or “sp.”) were considered. The resulting top 10 species were: *Aliarcobacter cibarius* (accession number: GCA_005818975.1), *Aliarcobacter cryaerophilus* (accession number: GCF_011045415.1), *Arcobacter acticola* (accession number: GCA_013177675.1), *Arcobacter suis* (accession number: CP032100.1), *Aliarcobacter butzleri* (accession number: GCF_000014025.1), *Bacteroides graminisolvens* (accession number: GCA_000428125.1), *Arcobacter caeni* (accession number: GCA_003063245.1), *Prevotella copri* (accession number: GCA_000157935.1), *Zoogloea ramigera* (accession number: GCA_006539865.1), and *Dechloromonas agitata* (accession number: GCA_000519045.1).

### 3.3 Pathogenic microorganisms detected in influent samples

**Figure 6:**
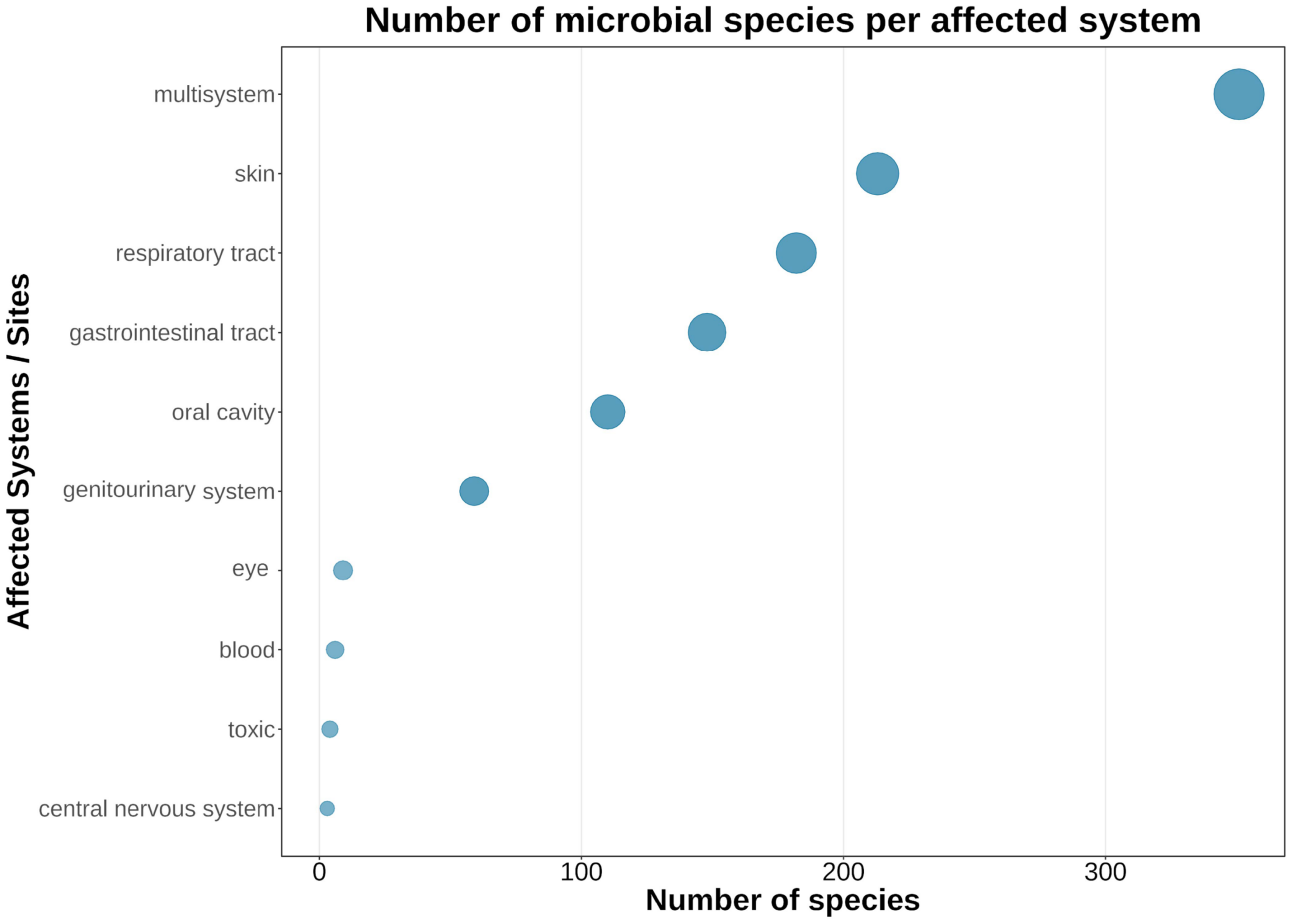
Number of microbial species (bacteria and eukaryotes) assigned to each primary affected system. The affected systems are listed on the x-axis, and the number of species on the y-axis.

A total of 1,085 pathogenic microbial species (853 bacteria and 232 eukaryotes) were detected across the six WWTPs. A complete list of all species together with their primary affected systems is provided in Supplementary Table S5. Based on their primary affected system (Figure 6), the largest categories were multisystem (351 species), skin (213 species), respiratory tract (182 species), and gastrointestinal tract (148 species). Other affected systems included oral cavity (110), genitourinary system (59), eye (9), blood (6), toxic (4), and central nervous system (3). Representative multisystem pathogens included *Acinetobacter baumannii*, *Pseudomonas aeruginosa*, *Klebsiella pneumoniae*, *Staphylococcus aureus*, and the fungus *Candida auris*. Skin-associated species comprised *Aeromonas hydrophila*, *Mycobacterium marinum*, *Staphylococcus epidermidis*, and the fungi *Trichophyton rubrum*, *Sporothrix schenckii*. Respiratory tract pathogens were represented by *Mycobacterium tuberculosis*, *Legionella pneumophila*, *Streptococcus pneumoniae*, *Bordetella pertussis*, and the fungi *Aspergillus fumigatus*, *Pneumocystis jirovecii*, *Histoplasma capsulatum*. Gastrointestinal tract pathogens included *Escherichia coli*, *Salmonella enterica*, *Campylobacter jejuni*, *Vibrio cholerae*, *Clostridioides difficile*, and the protozoa *Cryptosporidium* spp., *Giardia intestinalis*, *Entamoeba histolytica*.

### 3.4 Pathogenicity risk assessment

**Figure 7:**
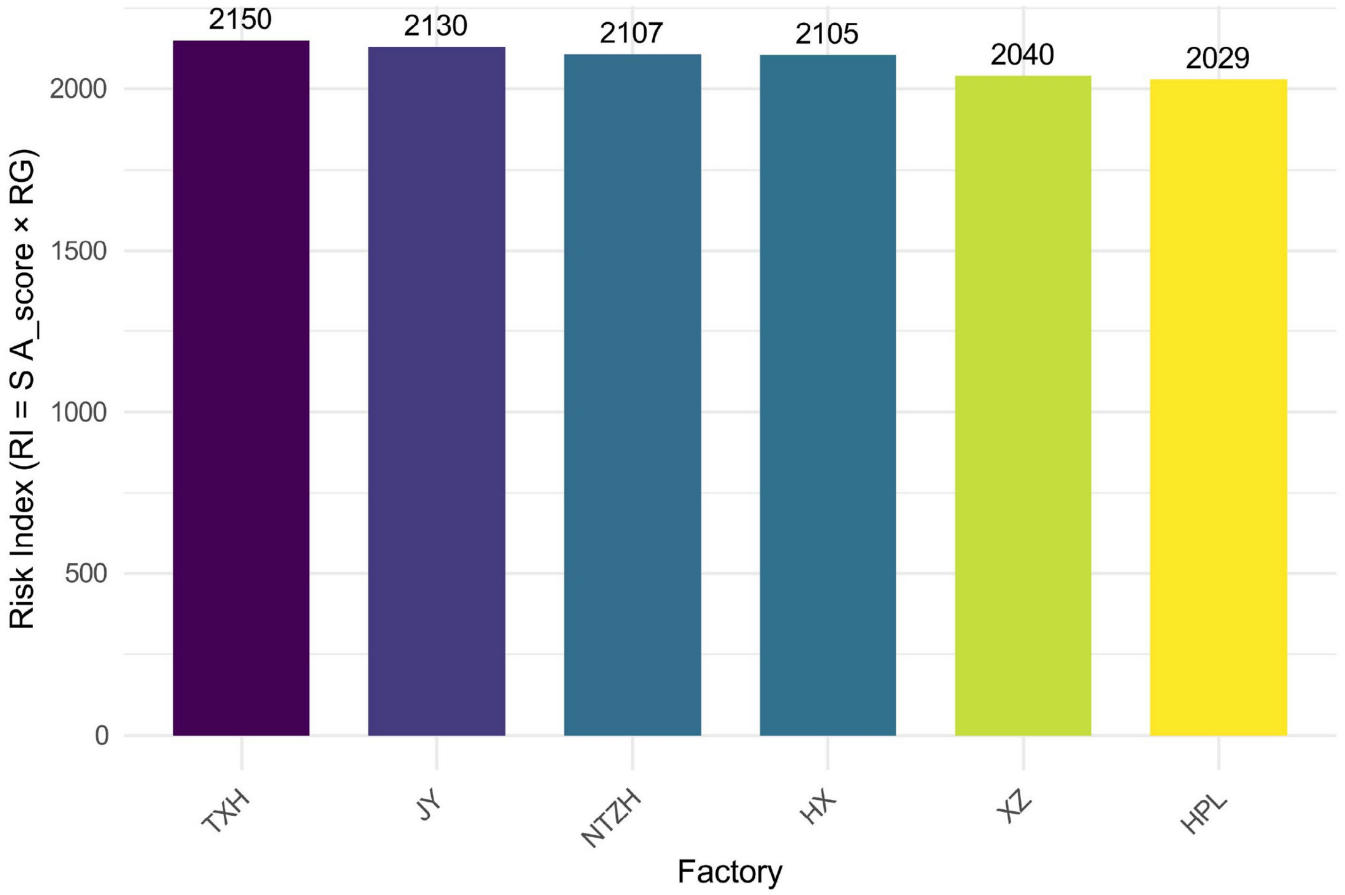
Exploratory hazard prioritization score (Risk Index, RI) for pathogenic bacteria in influent of six WWTPs. The bar chart presents the calculated Risk Index (RI = *Σ* abundance score × risk group, RG) for each WWTP. Y-axis: Risk Index value; X-axis: WWTP sampling sites. The RI values range from 2029 to 2,150. This order represents a descriptive comparison among the six facilities only; RI is an exploratory heuristic and has not been externally validated as an absolute measure of public health risk. Note: RI integrates the risk group (RG: 1–3) and abundance score (0–3) of all detected bacterial species. Higher RI in this dataset indicates a greater weighted contribution by abundance and risk group. XZ: Xinzhuang Sewage Treatment Plant; HX: Huaxi Sewage Treatment Plant; JY: Jinyang Sewage Treatment Plant; TXH: Tangxun Lake Sewage Treatment Plant; NTZH: Nantaizi Lake Sewage Treatment Plant; HPL: Huangpu Road Sewage Treatment Plant.

To evaluate public health risk beyond taxonomic presence and relative abundance, we performed a quantitative pathogenicity risk assessment. Each bacterial species was assigned a risk group (RG) based on established pathogenicity criteria (see Methods), and relative abundance was converted into an abundance score (0–3). A Risk Index (RI = *Σ* abundance score × RG) was calculated for each WWTP. The RI values for the six WWTPs ranged from 2029 to 2,150. Tangxun Lake WWTP (TXH) had the highest RI (2150), followed by Jinyang (JY, 2130), Nantaizi Lake (NTZH, 2107), Huaxi (HX, 2105), Xinzhuang (XZ, 2040), and Huangpu Road (HPL, 2029) (Figure 7). This order represents an exploratory hazard prioritization score for descriptive comparison across facilities and should not be interpreted as an absolute or externally validated risk ranking. The complete dataset, including each pathogenic species, its risk group (RG), and its abundance score in every WWTP, is provided in Supplementary Table S6.

**Figure 8:**
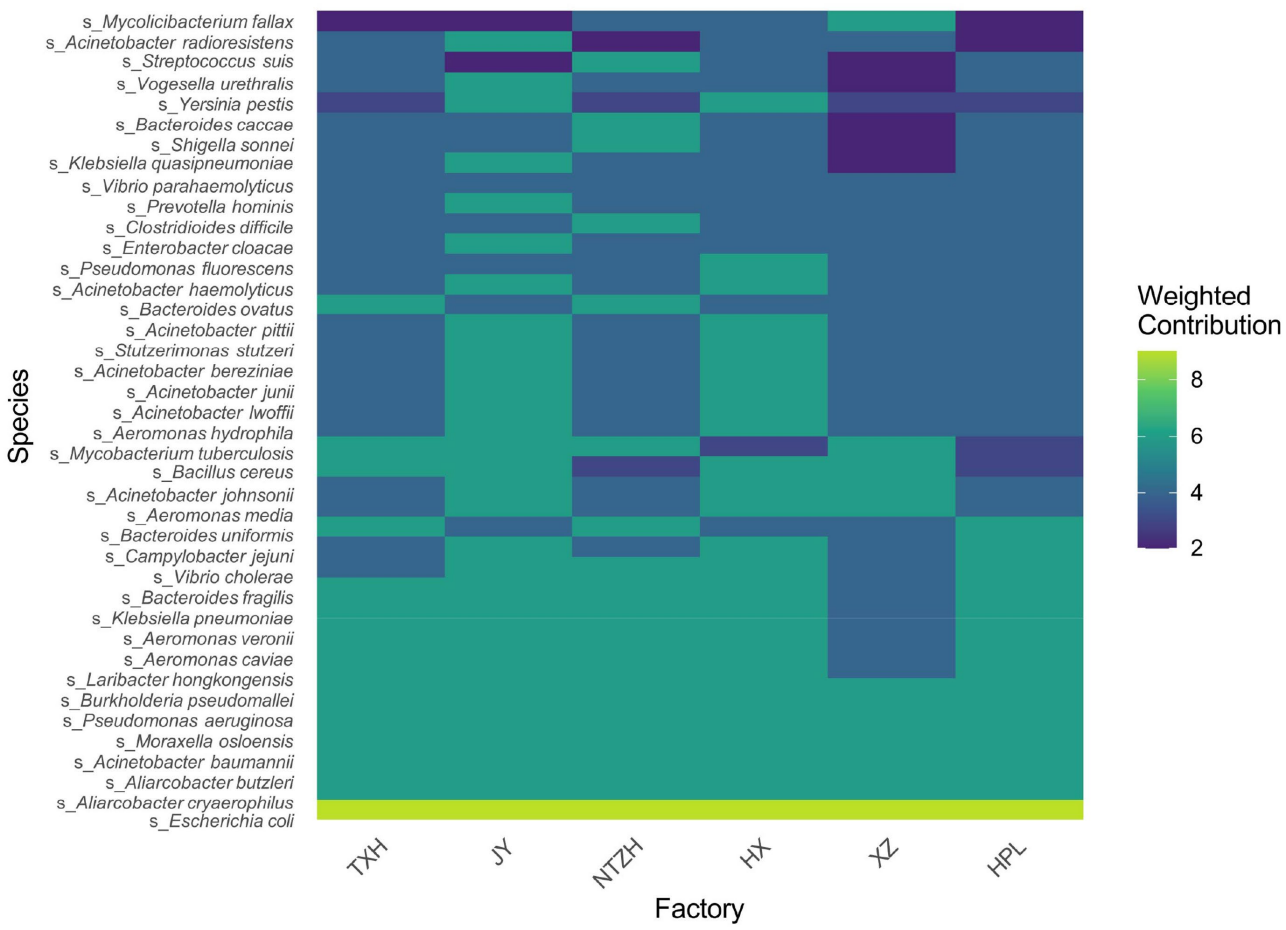
Exploratory hazard prioritization: top 30 species by weighted contribution per WWTP. Each cell represents the abundance score (0–3) of a given species in a given plant, converted from relative abundance as follows: 0 = 0%; 1 = ≤0.1%; 2 = 0.1–1%; 3 = > 1%. The Risk Index (RI) for each plant was calculated as RI = Σ (abundance score × risk group, RG), where RG values (1, 2, 3) were assigned based on DSMZ, BMBL (6th edition), the Chinese Catalogue of Pathogenic Microorganisms, and NCBI case reports. RI is an exploratory heuristic for internal descriptive comparison among the six WWTPs and does not represent absolute risk. Higher RI in this specific dataset indicates a greater weighted contribution by abundance and risk group, not a validated measure of absolute public health risk. Species shown are those with the highest weighted contributions (abundance score × RG) across all plants. XZ: Xinzhuang Wastewater Treatment Plant; HX: Huaxi Wastewater Treatment Plant; JY: Jinyang Wastewater Treatment Plant; TXH: Tangxun Lake Wastewater Treatment Plant; NTZH: Nantaizi Lake Wastewater Treatment Plant; HPL: Huangpu Road Wastewater Treatment Plant.

The heatmap (Figure 8) visualizes the weighted contribution (abundance score × risk group) of the 30 most important pathogenic species for each plant. *Escherichia coli* (RG = 3) was the highest contributor (weighted score = 9) in all six WWTPs. Many RG = 2 species, such as *Aliarcobacter cryaerophilus*, *A. butzleri*, *Acinetobacter baumannii*, *Klebsiella pneumoniae*, and *Pseudomonas aeruginosa*, each contributed 6 points in most plants. Despite a shared core of high-risk species, each WWTP displayed distinct features: HPL and NTZH were dominated by enteric pathogens (e.g., *Laribacter hongkongensis*, *Vibrio cholerae*, *Campylobacter jejuni*) with several RG = 2 *Bacteroides* species. TXH and XZ showed enrichment of respiratory and zoonotic pathogens, including *Mycobacterium tuberculosis* and *Bacillus cereus* (both RG = 3, weighted 6). HX and JY had a higher prevalence of *Aeromonas* species and also contained *Yersinia pestis* (HX) or *Mycobacterium tuberculosis* (JY). NTZH uniquely exhibited *Clostridioides difficile* (RG = 2, weighted 6), while XZ was notable for *Mycolicibacterium fallax*. The complete list of abundance scores, RG values, and weighted contributions for all top-30 species per plant is provided in Supplementary Table S7.

### 3.5 Antibiotic resistance genes (ARGs) detected in influent samples

**Figure 9:**
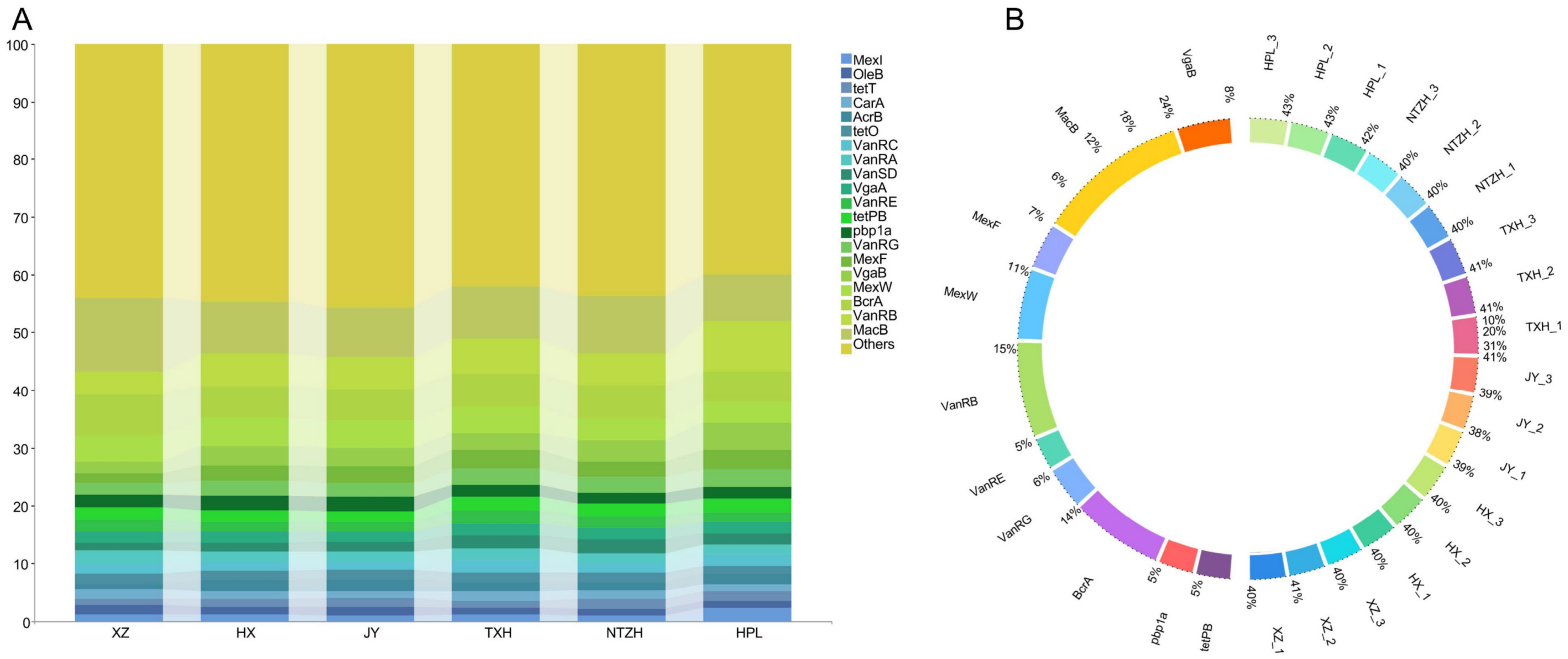
Analysis of antibiotic resistance genes at the wastewater treatment plant inlet: **(A)** Bar plot of the top 20 antimicrobial resistance genes (normalized by TPM) across the six samples. The stacked bars show the relative abundance and composition of key resistance genes; **(B)** Circos plot illustrating the composition of predominant ARGs. The numbers in the picture represent the relative proportions of different ARGs. XZ: Xinzhuang Sewage Treatment Plant, HX: Huaxi Sewage Treatment Plant, JY: Jinyang Sewage Treatment Plant, TXH: Tangxun Lake Sewage Treatment Plant, NTZH: Nantaizi Lake Sewage Treatment Plant, HPL: Huangpu Road Sewage Treatment Plant.

The ARG detections reported here are based on read-level homology to the SARG database (level a). We did not assess whether these reads constitute intact, functional ARGs (level b) or reside on mobile genetic elements (level c). A total of 357 antibiotic resistance genes (ARGs) were identified at the genus level, revealing substantial genetic diversity among the samples (Supplementary Table S8). Both the bar chart (Figure 9A) and Circos plot (Figure 9B) illustrated that although the samples originated from distinct locations (including XZ, HX, JY, TXH, NTZH, and HPL), the ARG profiles were consistently dominated by a “core resistome.” This core set was primarily composed of multidrug efflux pumps (e.g., *MexF*, *MexW*, *MacB*), *β-lactam* resistance genes (e.g., *pbp1a*), tetracycline resistance genes (e.g., *tetPB*, *tetT*), genes associated with vancomycin resistance operons (e.g., *VanA*, *VanC*, *VanE*). These conserved genes maintained high abundance across all sampling sites, collectively accounting for approximately 60% of the total ARG abundance (based on TPM normalization) on average. These findings further emphasize the fundamental role of urban wastewater as a critical environmental reservoir for the accumulation, maintenance, and dissemination of diverse antibiotic resistance determinants.

Correlation analysis revealed strong associations between MGEs and multidrug efflux pump genes, as well as selective co-occurrence between specific MGEs and genes associated with vancomycin resistance operons (*VanA*, *VanB*, *VanC*, *VanD*, *VanE*, *VanG*) or the beta-lactam target gene (*pbp*). However, as these correlations are based on read-level co-abundance from a limited sample size (*n* = 6) without multiple-testing correction, they should be interpreted as exploratory patterns rather than evidence of physical linkage or horizontal gene transfer, and warrant validation through long-read sequencing or functional studies (Figure 3).

These findings demonstrate the ubiquitous presence of specific—and often multidrug-resistant—ARG profiles across the six WWTPs, thereby underscoring the significance of wastewater environments as pivotal reservoirs for the persistence and dissemination of clinically relevant antibiotic resistance mechanisms.

## 4 Discussion

### 4.1 Unveiling the core wastewater microbiome: global dominance of Arcobacteraceae and its persistence through treatment processes

The dominant phyla identified across the studied WWTPs were Proteobacteria (35.28–67.71%) and Bacteroidota (10.99–20.08%), consistent with prior reports on surface water and wastewater systems (Becerra-Castro et al., 2016). Proteobacteria has been recognized as a ubiquitous dominant phylum in wastewater treatment systems. A global survey covering 74 cities across 60 countries revealed that this phylum is present in approximately 70% of wastewater samples, constituting a stable core microbiome (Yan et al., 2025). In China, Proteobacteria and Bacteroidota are the dominant phyla in both sewage and activated sludge, with relative abundances of 57.57 and 12.23% in Yuxi, Yunnan Province (Jia et al., 2025), and 46.2–62.9% and 14.8–21.8% in high-altitude WWTPs in Tibet (Zhang et al., 2025), respectively. These phyla are ubiquitous in aquatic environments, display high metabolic versatility, and respond rapidly to nutrient-rich conditions, establishing them as a “universal core microbiota” in sewage ecosystems (Shin et al., 2015; Singh et al., 2023). These discrepancies are likely attributable to differences in influent characteristics, treatment processes, climatic conditions, and methodological approaches.

The top 10 most abundant species were predominantly members of the Arcobacteraceae family, occupying six of the ten positions. This dominance is not an isolated observation but a globally conserved feature of wastewater influents: Arcobacteraceae have a global pooled prevalence of 69.2% in aquatic environments, with the highest prevalence occurring in raw sewage and WWTP influents (Venâncio et al., 2022). Arcobacter has been consistently reported as a dominant genus in WWTP influents across multiple studies in China, with relative abundances ranging from 6.79 to 67.90% of total reads (Zhao et al., 2024; Zheng and Wen, 2019). Notably, several of these species—*A. cryaerophilus* and *A. butzleri*—are recognized as emerging human pathogens of animal origin (Savelli et al., 2025). And critically, Arcobacter cells do not flocculate or attach well to activated sludge flocs, remaining dispersed in the water phase and thus persisting through treatment process (Kristensen et al., 2020). Taken together, the consistent dominance of Arcobacteraceae across our six plants aligns with global and Chinese surveys, reinforcing their status as a core influent microbiome component, while their treatment persistence and pathogenic potential underscore the value of plant-specific influent profiling for surveillance prioritization.

### 4.2 Observed pathogen profiles and their potential public health implications

The pathogen profiles detected in this study show both commonalities with and differences from previous wastewater surveys. The enteric pathogens enriched in HPL and NTZH (*Vibrio cholerae*, *Campylobacter jejuni*) have been consistently detected in wastewater from South Asia and other regions where these waterborne pathogens are endemic (Street et al., 2025; Sthapit et al., 2025); in China, *V. cholerae* has also been detected in urban wastewater with seasonal variation (Zhang et al., 2025); The detection of *Mycobacterium tuberculosis* in TXH, XZ, and JY is consistent with its detection in wastewater from multiple countries, including the United States, South Africa, Poland and Germany (Paulos et al., 2026; Mtetwa et al., 2022a; Mtetwa et al., 2022b), suggesting that influent pathogen profiles may reflect local disease ecology. Furthermore, opportunistic fungal pathogens including *Aspergillus fumigatus* was detected across the studied WWTPs—a finding consistent with wastewater surveillance studies in South Africa, Mexico, and Austria (Assress et al., 2021; Gutiérrez-Anzueto et al., 2024; Haas et al., 2002). While these fungi pose limited risk to immunocompetent individuals, they can be fatal to HIV patients, organ transplant recipients, and chemotherapy-treated cancer patients (Köhler et al., 2017). In WWTPs where enteric pathogens were enriched, such as Huangpu Road (HPL) and Nantaizi Lake (NTZH), chlorine-resistant parasites (*Cryptosporidium* spp., *Giardia intestinalis*) were also detected, *Cryptosporidium* spp. and *Giardia intestinalis* are difficult to remove by conventional disinfection (Xiao et al., 2018). However, we did not assess pathogen viability, exposure routes, or the actual risks associated with water reuse. Therefore, no specific intervention recommendations are made based on these data.

Although detected at low abundance, rare but severe pathogens such as *Plasmodium falciparum*, *Babesia*, and *Naegleria fowleri* (“brain-eating amoeba”) were also present. Their detection is noted. Their presence in wastewater influents is noteworthy and suggests that continued surveillance may be warranted, but this does not imply immediate public health action (Ekici et al., 2022; Fang et al., 2015; Kamana and Zhao, 2023). The RI (abundance score × risk group) ranged from 2029 (HPL) to 2,150 (TXH). *E. coli* contributed the most in all plants. Plants with enteric pathogens (HPL, NTZH) had lower RI, whereas TXH—enriched in both enteric and respiratory pathogens—showed the highest value (2150). However, this RI is an internal descriptive heuristic: it has not been externally validated. It should not be interpreted as an absolute public health risk ranking.

### 4.3 Antibiotic resistance genes in influent samples: core resistome and MGE associations

This study identified multidrug efflux pumps (e.g., MexF, MexW, MacB), *β*-lactam resistance genes (e.g., pbp1a), tetracycline resistance genes (e.g., tetPB, tetT), and genes associated with vancomycin resistance operons (e.g., VanRA, VanRC, VanRE) as core and widespread antimicrobial resistance genes (ARGs). These findings align with studies from Africa, Asia, and South America (Hendriksen et al., 2019), and with global metagenomic surveys showing that efflux pumps and β-lactamases are dominant ARG types in WWTPs worldwide; ARG distribution varies regionally, with elevated prevalence in some African and Asian regions compared to Europe and North America (Zhu et al., 2025). Within China, nationwide surveillance identified a core resistome of 117 ARGs accounting for 69.6% of total ARG abundance, confirming the representativeness of our results at a national scale (Wang et al., 2025). This pattern may reflect differences in antibiotic consumption and wastewater catchment characteristics—though these factors were not directly measured in our study.

The MGE-ARG correlations observed in our dataset—particularly the strong associations between MGEs and multidrug efflux pump genes—are consistent with a nationwide survey of 28 Chinese WWTPs (Liu et al., 2026) and a Peking University-led study on urban rivers (Wu et al., 2022), both of which identified transposases as key drivers of ARG dissemination. Internationally, metagenomic analyses of WWTPs in Morocco and across multiple geographic locations have highlighted the role of MGEs in ARG dissemination (Wardi et al., 2024; Garner et al., 2024). Notably, the selective MGE correlations observed for vancomycin resistance genes (*VanA*, *VanB*, *VanC*, *VanD*, *VanE*, *VanG*) and the beta-lactam target gene (*pbp*) further support the involvement of MGEs in the co-occurrence of diverse ARG types in municipal wastewater (Sanderson et al., 2020; Ju et al., 2019). Nevertheless, given the limited sample size (*n* = 6) and the lack of multiple-testing correction, these correlations should be interpreted as exploratory patterns rather than evidence of physical linkage or horizontal gene transfer, and warrant validation through long-read sequencing or functional assays.

### 4.4 Study limitations and future perspectives

Several limitations of this study should be acknowledged. Regarding study design: Seasonal confounding: The samples from Wuhan (October 2024) and Guiyang (February 2025) were collected in different seasons due to project scheduling and site access constraints. Because this study is purely descriptive and no cross-regional statistical comparisons are performed, each WWTP is described independently, and seasonal differences do not compromise the plant-specific conclusions. Nevertheless, the seasonal mismatch is a limitation, and future studies with synchronized multi-season sampling are required to separate seasonal from geographic effects. Pseudoreplication: Three grab samples were collected from the same location within each WWTP on a single day. These are technical replicates, not independent biological replicates. The true independent replication unit is the WWTP itself (*n* = 6). Descriptive statistics are reported without inferential tests (Hurlbert, 1984). Inlet-only sampling: This study focused exclusively on influent, providing baseline data on incoming hazards. We did not track the fate of pathogens or ARGs through the treatment process, so removal efficiencies and final risks remain unknown. Regarding data generation and analysis: Viability not assessed: Metagenomics detects DNA and cannot distinguish viable from non-viable microorganisms. Thus, the presence of pathogen or ARG reads does not indicate infectivity or functional resistance (Shen et al., 2022). Species-level identification uncertainty: Short-read taxonomic classification may produce false positives, particularly for certain fungi (e.g., *Coccidioides* spp.) and protists (e.g., *Naegleria fowleri*). No orthogonal validation (e.g., PCR) was performed. No negative controls: Sterile water or extraction blanks were not included; therefore, low-abundance detections could be influenced by contamination. Host read depletion: Human reads were not removed. Strain-level resolution not achievable: Short-read sequencing does not allow us to distinguish whether detected species (e.g., *Pseudomonas aeruginosa*) are represented by a single dominant strain or multiple coexisting strains. Future perspectives: Metatranscriptomic analysis on the same samples would help identify actively expressed ARGs and virulence factors. Multi-season sampling, long-read or Hi-C sequencing, and inclusion of process train samples would provide a more complete picture of temporal dynamics and actual public health risks. Future research with a larger number of WWTPs and multi-factor statistical modeling could explore synergistic or antagonistic effects between topography, anthropogenic activities, and infrastructure on microbial hazard distribution – an aspect not addressed in our descriptive baseline study.

## 5 Conclusion

This study provides a descriptive metagenomic characterization of biological hazards in influents from six WWTPs. A wide range of pathogenic bacteria (853 species) and eukaryotic microorganisms (232 species) were detected, with enteric pathogens enriched in some plants (e.g., Huangpu Road, Nantaizi Lake) and respiratory pathogens in others (e.g., Xinzhuang, Jinyang, and Tangxun Lake). A core resistome—comprising multidrug efflux pumps, *β*-lactamases, and tetracycline resistance genes—was present across all six plants, accounting for approximately 60% of total ARG abundance. Correlations between MGEs and ARGs were observed, but do not confirm horizontal transfer. These findings demonstrate that WWTP influents harbor distinct plant-specific hazard profiles, and that catchment characteristics and treatment processes are associated with variations in microbial and resistome composition. This descriptive baseline provides a foundation for data-informed surveillance prioritization, rather than prescriptive interventions. Future studies incorporating longitudinal sampling, viability assessment, and long-read sequencing will be required to translate such baseline data into actionable public health strategies.

## Data Availability

The datasets presented in this study can be found in online repositories. The names of the repository/repositories and accession number(s) can be found in the article/Supplementary material.

## Footnotes

1
https://bioinformatics-centre.github.io/kaiju/downloads.html

2
https://galaxyproject.org/use/args-oap/

3
https://card.mcmaster.ca

4
http://ardb.cbcb.umd.edu/

5
https://www.ncbi.nlm.nih.gov/mesh

6
https://www.ncbi.nlm.nih.gov/taxonomy
